# Juvenile Delinquency

**Published:** 1963-04

**Authors:** R. F. Barbour


### JUVENILE DELINQUENCY.

### A Family Practitioner's Problem?*

BY

R. F. BARBOUR.

The title of this paper includes a question mark because I am not at all sure how far family doctors intend to enter the field of social medicine, or even whether the community wishes them to. Let me quote from a recent editorial in the Lancet commenting on the Presidential Address at the B.M.A. meeting last summer: "Mr. Fraser emphasised the need, above all, to face some of the problems of today, increasing leisure, alcoholism, juvenile delinquency, and road accidents, and he thought that the State and public bodies should take a more practical part in their solution. His implied acceptance of the need for partnership between the State and the profession in social planning is something which is by no means universally accepted."

In situations of equal stress it is often constitution, influenced by early upbringing, which decides whether one acts out or inhibits; the former may end in losing one's temper, the latter may result in increased peristalsis or indigestion. Either physical symptoms or anti-social behaviour may arise if one is in a frustrating situation. The boundary line between naughtiness and illness is not the clear thing our fore-fathers seemed to think. Later, I will give instances of how a child will alternate between soiling and stealing, depending on the reaction of the parents. Before discussing individual cases, however, let us look at the nation-wide pattern of juvenile delinquency, and then at the Bristol picture.

### DELINQUENCY IN ENGLAND AND WALES AND BRISTOL

Statistics are issued annually by the Home Office giving the numbers of children (aged 8–14) and "young persons" (aged 14–17) found guilty of offences of all kinds. No figures are available for those below the age of 8 because in English law no child under that age is capable of criminal intent. These figures show a general increase in crime in recent years, particularly amongst boys, the peak age being 14. Traffic offences now constitute over 60 per cent of all crimes, and there has been an increase in drunkenness, violence, and theft. Convictions for theft rose by 62 per cent between 1955 and 1961, but not all forms of theft show the same degree of increase. Larceny from automatic machines is doubled; theft of motor vehicles, although small in absolute numbers, is trebled; larceny from motor vehicles is up by 80 per cent.

There is not complete agreement about these fluctuations. Before the War, poverty and distress were usually given as the main causes of crime. After the War it was considered that rationing and the unsettled home life were largely responsible; we now tend to blame increased temptation in the form of self-service stores and the increased number of cars parked unattended, frequently unlocked. It is also undoubtedly true that offences by children are now more readily reported to the police, for while the community used to take responsibility for what was happening in its midst, it is now the practice to "call in the Law". Police practice has also changed; but it should be noted that in 1959 a further 16,000 children (20 per cent) were cautioned by the police and not prosecuted.

Now let us come nearer home and consider the Bristol figures. These show a similar pattern of increasing crime. The number of offenders in the last year increased

* Based on an address to the Bristol Medico-Chirurgical Society, 12th December 1962

by 16 per cent, with the bigger increase in the lower age-group, 8–14 years. Crime among girls increased by 72 per cent (notably shop-lifting in departmental stores) and among boys by 10 per cent. But despite these most uncomfortable figures, our figure is well below the national average and considerably lower than in most other large towns. The risk for any boy is still below 1 per cent. Just as we are not sure why the national figures fluctuate, so we are not sure why our record is—so far—so good.

In 1951 the Carnegie Trust decided to carry out a survey of Juvenile Delinquency as a preliminary to possible action. John Mack carried out the Survey and his Report entitled "Family and Community" was issued in 1953. After looking over the country as a whole he suggested Bristol as a possible field for action. His reasons for suggesting Bristol are interesting. Bristol was considered "to be more likely than most cities of comparable size to suggest the shape of the social problems of the future". After commenting on the relatively low figure for delinquency in 1948 he goes on "It is probable that Bristol's low rate truly reflects its comparatively favourable environment. The city avoided the development of nineteenth century heavy industry. Its population increase was even and unhurried, compared with the towns of the industrial North, so that there was no drastic sacrifice of amenity—the standard of living is comparatively high. The development of new industries, notably aircraft, has increased immigration and population mobility but it has also sustained the standard of living. Bristol with little of the débris of the Industrial Revolution to clear up and well launched on the Supersonic waves of the Neo-technical Revolution, is a better prototype of the city of the future than are the urban concentrations of the North.

In the second place the people concerned with juvenile delinquency in Bristol are sympathetic towards the idea of social experiment. There was already a high degree of informal integration among those dealing with delinquents. All this suggested that such juvenile delinquency as existed in Bristol was not a consequence of undue slackness in the organisation of the relevant services—the increase in juvenile delinquency in Bristol, as elsewhere, was due to forces beyond the control of the very competent people in charge of the Court and its allied services."

As a result of Mr. Mack's Report the organisation that became known as the Bristol Social Project was set up. I will return to some of the Project Team's recommendations later.

### CAUSES OF DELINQUENCY

What are the present views regarding the causation of juvenile delinquency? Most writers list factors under four headings:

1. Constitutional.
2. Early conditioning or training.
3. Family and community patterning.
4. Trigger factors.

### *Constitutional Factors*

It is, at present, extremely difficult to distinguish genetic factors from early brain damage—when genetically similar pairs of monovular twins are available there are few separated early enough to compare the effects of dissimilar environments. Probably the best known recent work in this country is by Slater who wrote "genetic factors play a considerable part in the development of personality but the appearance of overt symptoms and the breakdown of adaptation are largely environmentally caused".

The relationship of brain damage to later asocial traits has often been noted, but not all cases of, say, encephalitis develop psychopathic traits. Of recent years much attention has been focussed on disturbances of the temporal lobe and their relation to emotional outbreaks. There is now general agreement that the limbic system is the neurological substrate of emotion. Lesions in the hypothalamic nuclei, temporal lobes, or the medial frontal cortex are liable to produce personality disturbances.

Pasamanick in the United States matched normal children with those who had behaviour disorders and found significantly more complications of pregnancy and delivery and also prematurity in the latter. "Non-mechanical abnormalities such as toxaemia appeared to be the important factor, rather than the mechanical factors of delivery—these associations were still present even when intellectual and environmental factors were controlled. Hyperactive, confused and disorganised children have even more of these abnormalities in their background". These findings were not however strongly supported by Brander when he re-examined the children in the Newcastle 1000 Family Study—he did agree though that minor disorders of personality, perception and co-ordination were associated with residual effects of asphyxia at birth. Histories taken at approved schools show that compared with the normal population delinquent boys are more likely to have had peri-natal problems. In 1953, 100 consecutive admissions to Kingswood Classifying School were studied, and 84 per cent were found to have E.E.G.s of a degree of abnormality found only in 5 per cent of non-delinquent juveniles. In only 5 cases, though, was the form of the E.E.G. similar to that associated with a recognised clinical syndrome. The remainder all showed "theta activity," and 68 displayed poorly localised slow activity of the "delta" type. There are, however, plenty of "reproductive casualties" (to use Pasamanick's phrase) who do not later show delinquent tendencies. Something else must be necessary. Recently, Pond and Gruneberg compared three groups of children, epileptic with behaviour problems, epileptic without behaviour problems, and non-epileptic with behaviour problems, and concluded that background factors independent of the epilepsy were mainly responsible for the behaviour difficulties. Put more simply, if an epileptic child has a really understanding family, a mother who is able to love him as an individual, and a father who will help and encourage him, despite his handicap, there will not be behaviour problems.

*Early conditioning*

Medicine has always been concerned with the starved baby, the undernourished child. It has long appreciated how markedly a tumour of the brain or encephalitis could affect developing personality, but it is only in the last half-century that we have learned the many and varied ways in which the personality of a child can be distorted through lack of adequate sensory stimulation. We now know that some children who were classified as grossly defective were, in fact, totally deaf and that their lack of intelligence was due to their brains not receiving the every-day normal stimuli which could be synthesised into sensations, concepts, ideas. Output will not be normal unless there is the appropriate intake. Sense organs need to have adequate stimuli in quality and quantity. The natural urge of the child, as of all animals, is to form connections with, be affected by, the things, the people in the world around. A world unseen, as for a blind child, can be learned about by touch and sound, but it is essentially different, the signals for danger in the world of sound are not usually recognised by the child with sight. We are just beginning to appreciate the importance of the phase in development where touch is the main means of communication. To be touched by a thing and by a person can be two different experiences, though we may be touched by a person as if we were a thing. If a child is not touched, is not played with, its personality may be affected.

We speak of the emotionally deprived child. It is with parents that a child first experiences an intimate give-and-take relationship, call it love if you like; if this relationship is pleasurable it will be sought after, at first with parents, later with others. If on the other hand it is not satisfying, or worse still it is off-putting, inhibiting, then anxiety can be aroused and later relationships will be similarly coloured. If mother is warm, consistent, predictable, then this is the pattern evoked in the child. If mother starts this way and then later rejects, the resulting behaviour is not unlike that of the animal which makes for the food box but receives a painful stimulus en route—it becomes uncertain, wavers in its performance, later may become indrawn, inhibited. Erickson has shown how in the second and third years a child is struggling to assert autonomy, in the fourth and fifth to develop initiative—if the environment is not receptive, helpful, then the whole later development of that individual is affected.

Let me say here that there is very likely to be disagreement among fathers as to what the ideal child should be like. Where do we, as parents, draw the line between independence and obstinacy? Was it the spider or Robert the Bruce's permissive mother who encouraged him to go on trying seven times?

Unloved children, not getting the proper intake of affection, will be themselves affectionless in later life. Children exposed to marked swings with consequent unpredictability in their parents' reactions, are likely themselves to fail to develop emotional stability.

*Family and community training*

In our culture it is the parents who train us socially, who make us either "conformers" or "non-conformers". By the age of five our own personal ratio of dependence and independence has been set up. Rules are things which we try to ignore, compensate for, or, on the other hand, are things which we keep. Grown-ups are reacted to as being predictable, fair, or as people to fight and, if possible, elude.

The techniques used by grown-ups to achieve their ends may be chiefly punishment or withdrawal of approval. The efficacy of the methods depends in part on consistency—in part on the degree of attachment already developed between parent and child and the presence of other grown-ups by whom the child may be corrected or to whom he can turn for consolation. The emotional atmosphere in an extended family is different from that existing where the home consists only of parents and siblings. Parents also vary in their ability to make clear to the child what he is expected to learn. A child punished for taking bananas one week was caught taking apples the next. His reply on being told off was that "you told me not to take *bananas*". Or again, in punishing a child who has taken money from her purse, the mother may say "you must never do that again", leaving it to the child to identify that it is the taking money without asking that is wrong, that it is still permissible to take money if mother says when she sends him to a shop, "you will find the money in my purse". It requires a fair degree of intelligence on the child's part to see what the underlying factor is in money stolen at home, shop lifting, and bringing home without permission a cousin's toy.

The difference between "perks", "scrounging" and stealing may be obvious to the adult though not to the child. Taking money or stamps is clearly always wrong but father may come back with office stationery. Twenty minutes is one span of time when it is what a child is allowed for going to a shop, quite another when father leaves his car in a limited parking zone.

By the time a child is seven, his main personality characteristics are well established. The Gluecks have picked out the characteristics that they consider "pre-delinquent"—adventurousness, stubbornness, suggestibility, emotional instability; also defiance, social assertion, suspiciousness, destructiveness—these terms are used in certain well defined ways—but there are also social factors which need to be assessed. They also have picked out certain family traits and the combination of these qualities in the child with those in the parents which tend to produce the child who is likely to act asocially.

So far we have mentioned only the factors which apply to any child in any culture. Whether he, in fact, becomes an official delinquent depends entirely on what are the present laws of the land. Any child is also much affected by the attitude of the other members of his family and of the community to the interpretation of laws and to law-breaking. Regulations may be one thing, laws another. A police court summons for a traffic offence usually makes one an object for commiseration; if traffic laws are considered to be something to evade, this attitude soon spreads to other laws, something to be interpreted rather than to be obeyed.

### *"Trigger" factors*

Finally there is the "trigger" factor which in itself may be relatively unimportant—frustration, disappointment, boredom in a maladjusted individual can lead to taking chances, showing off, compensating oneself in a material way, any of which may result in a delinquent action, and then even such apparent irrelevances as the passing of a patrol car, a policeman's indigestion, or a neighbour's irritability, may, in fact, decide whether the boy appears in Court.

If children in approved schools have a high proportion of atypical cerebral rhythms, it is equally true that they come from disturbed homes. A recent survey at Kingswood Classifying School showed over 80 per cent as coming from homes where there was a high incidence of quarrelling or separated parents, breakdown or alcoholism. The delinquent population also does not have a normal I.Q. distribution. It is loaded on the dull side.

### THE ROLE OF THE FAMILY DOCTOR

And how does all this affect family practice? As many of you know, we see at the Bristol Child Guidance Clinic a large number of cases for the Juvenile Court. Last year there were 103. I thought it would be of interest to find out how far family practitioners were "in the picture". So we sent out a pro-forma in 25 consecutive cases. In only 9 cases did the doctor know that his patient had been before the court. In half of the cases he felt that he could have given me relevant information for my report to the Court, but in only half. One is led to the conclusion that the family doctor is not sufficiently frequently consulted in problems of this kind.

Is this an area where preventive measures are not being adequately used? In 1960 the Second United Nations Congress on the Prevention of Crime and Treatment of Offenders was held in London. Dr. Gibbens, on behalf of the World Health Organisation, presented a report on New Forms of Juvenile Delinquency, their origin, prevention and treatment. Under Preventive Policies the first listed is Medical Services. He writes "The prevention of maladjustment originating in the pre-school period often falls upon the medical services. In the past, they have often been concerned mainly with physical health, but it is now recognised that their training should enable them to detect family situations which have a bearing upon mental health. At a later stage, school medical officers are well placed to observe the first signs of emotional as well as physical disturbance, and their enquiries can proceed in the framework of medical confidence, which may be reassuring to the parent." The family doctor is not mentioned!

The Lancet of the 3rd November 1962 had an "Annotation" entitled "The Family Doctor". It read: "At the same time as medicine becomes more complex, so the patient's need for a real family physician increases. He needs a physician who will look after the whole man, see him against the background of his family, his home and his work, interpret him to a consultant when necessary, and the consultant to him."

I personally am in full agreement with this sentiment and I would go further and say that at times the infant, toddler, or child needs a physician who will interpret him not only to the consultant, but also to his parents.

The thesis I am putting forward is briefly this. The family doctor has accepted the role of interpreter where nutritional needs or sensory defects and handicaps are concerned. Can he stop short when it is a question of emotional needs? Possibly so if he limits his field to the curative, but not if he is concerned with the overall health of the child. We recognise inborn defects of metabolism which require special diets. Are there children with other constitutional handicaps on whom we should keep a special check, and if so, which? I would suggest that there are, and that we should consider:—

*Firstly*—Children who have had perinatal handicaps; babies whose mothers have been toxaemic, babies with evidence of anoxia or jaundice.

*Secondly*—The child who has had febrile or teething convulsions, again evidence of faulty cerebral structure. They do not usually become epileptics in later life, but a high proportion seem to act as much younger children, very explosively, rather like petrol compared to paraffin. If the mother can cope easily then in a few weeks the problem ceases. Often, however, the mother becomes worried, or the child's aggression upsets her emotional balance and she storms and then the child starts wandering off and we get the typical pre-delinquent pattern. Admittedly this child taxes the emotional resources of any home. The barbiturates often make these children worse, amphetamine helps about one in four, so does Moditen which has the advantage of being turned out as an elixir 0.1 mgm. t.i.d. for a 3 to 4 year old.

*Thirdly*—There is the child who is slow to develop a dominant cerebral hemisphere, often late in talking. But to begin with he is neither clearly right- nor left-handed. Frequently the result appears as clumsiness and he seems to be specially prone to emotional tantrums. Here again the parent can be warned that their child is trying to meet life with a central nervous system organised differently from that of most children. Give him time and he will settle down but don't think that he is deliberately spilling the water.

Another group are the hyperkinetics, over-active children, who will not settle at anything—normally poorly co-ordinated, they are extremely inquisitive and attention-getting. In many of these as Pasamanick mentioned, there is a history of difficult birth.

All these are children constitutionally different from the average who require special techniques at the time they are learning that they have to conform. Man has been called a social animal, he has to learn to co-operate, but, as in everything else in life, the method of teaching as well as the content influences the result. In our culture, battles of will are likely to take place over eating and toilet training, later over sleeping habits and cleanliness. Many parents need advice especially if they have a difficult child after, say, two easy ones.

A not infrequent referral to the child guidance clinic is the encopretic. Usually mother says they have tried everything—kindness, harshness, making him wash his dirty pants, bribery—but by the time they come the child and his symptoms are clearly dominating the mother. The child, as he grows up, particularly if his "smelliness" is noticed at school, will often change to some other "nonconforming" symptom, the commonest of which is stealing. One of the problems in the treatment of the encopretics is that if you can get the mother to be more relaxed the child then becomes more boisterous, and there is a phase of six or eight weeks when the boy may be extremely difficult. If the mother can't take it, and again puts on pressure, the symptom returns; but if with support that phase can be worked through, the whole tension situation eases.

When taking the history of these young children one is trying to see how far the problem is constitutional, how far environmental. A pretty normal child with an obsessive mother can develop as much stress as an atypical child with a quite warm mother. When a mother refers to a child as hardened or says "he would not let me mother him", if she says she had thought of having him adopted, then one knows that the state of tension must have got to a point where the child's personality will almost certainly have been adversely affected.

Other critical points are the jealousy situations. In families where the space between children is about 3 years, just as the child is learning to be independent, the mother may become unpredictable owing to the ensuing pregnancy. Here is an opportunity for the father to come more into the picture. Again, if one child needs considerably more time and care because it is a spastic, or has, say, severe dermatitis, the next child may feel left out unless the father or other adult can compensate for the mother's relative lack of attention. Prolonged separation of mother and child, for whatever reason, tends to result in a difficult period of readjustment when they come together again. It is hard on a young child if his mother returns home "convalescent", he can't understand why mother can't pick him up and play with him as she used to, to him it seems like being rejected. Family moves, death of grandparents, illness of parents, are all times when emotional tensions are likely to rise and if the child has failed to become adequately secure at a younger age, delinquency may result.

In fact, if for any reason a child is making extra demands and they are not met, a degree of deprivation results. Some of the ways in which they may happen have been mentioned above. The mother may be absent, unpredictable, or labile. Mothering may also be inadequate in homes and institutions where to a child it may seem that he has several different mothers during the day.

The family practitioner is the person on the spot. He is likely to be in the home at times of crisis and although his primary interest may be in one patient, he can be aware of possible effects on other members of the family and, if necessary, he can advise. This approach is the standard one when the problem is an infectious disease; can it be extended to states of stress? It is probably on this preventive side that the family doctor has the biggest influence.

In my small survey, in only six of the twenty-five cases had the family doctor been consulted about behaviour difficulties in the child in question, and I think in only two of the six about the problem that led to the child appearing in Court. Does this indicate that parents do not consult their G.P.s about this kind of problem or that G.P.s have little constructive advice to offer, or possibly a bit of both?

*The Problem Child*

If at all possible, any child with a behaviour problem should be seen alone. Most children over the age of five or six will separate fairly easily from their mother; with only one person, the child, in the room, assessment is much easier, the child stops looking to mother for the answer, equally she cannot cut him short. I have already described the hyperkinetic child who lacks concentration, who flits from one thing to another. These things indicate the child who will need the maximum of help that the community can give.

If any naughty child is brought to one, the diagnosis is only complete when one understands the psychodynamics of the behaviour. No child wants to be unpopular or punished; if he still persists it can only be because somehow, to him, what he is doing is preferable, even if it is the lesser of two evils. Young children under the age of 10–11 often don't really know what the problem is, they only know they are unhappy or frustrated. Their drawings, their stories, their play, usually give pointers as to where the problem is. Admittedly to elicit this information may take 10–15 minutes and many family practitioners may feel they have not the time for this kind of enquiry.

Some children want the thing that they steal, say sweets or fruit. If a child takes money, the important question is, on what is the money spent? Some children take things to buy "affection", if you can give things away then other children will play with you. One pathetic boy whom I saw, stole a football from Woolworth's and he explained he thought that if he had a football then other boys would play with him. Another child with insufficient pocket money stole from Lewis's so as to be able to give his mother a birthday present. Some children comfort themselves with material things when they find themselves misunderstood; possessions are a substitute for affection. Occasionally a child only takes things from one person—possibly a step-mother—that may be to annoy, or sometimes it is as if they feel that if they have the belongings of a person they will become like that person. In other cases its a battle of wills. "You hide the gas money and I will prove that I can find it."

Then there are the children who persistently light fires—usually with an urge for power or to "make their presence felt". Often they are children who do not get enough attention for doing right and proper things.

In part, some of the increase in sex problems arises through the much greater frequency of one-sex families. When parents had families of four, five or more children usually both sexes were represented. Now, when families have only one or two children, the chances of both being of the same sex are quite high. This creates a need for planned opportunities of learning about anatomical and physiological differences between the sexes.

Children often work a 35-hour educational week. The school situation may compensate or aggravate the child's emotional difficulties. If a boy is of reasonable intelligence then he may get sufficient praise in school to compensate for indifference at home; if on the other hand he is dull, backward, and feels "rejected" at home and "unwanted" in school, then the chances of delinquency rise considerably.

There is always some pattern or underlying "meaning" in the delinquency. Punishment without understanding will only change the symptoms. Certainly punish, but also help the child with the situation which precipitated his delinquent act; his boredom, loneliness, lack of affection.

*Whose Responsibility?*

We return to the question mark: Are these delinquents our job? I suggest that it is very difficult to say they are *not* our job. The B.M.A. report on Adolescence a few years back referred to the need for guidance of the parents, for the prevention, early detection and correction of disharmonies in the home. If it is good medical practice to prescribe indefinitely for a diabetic or an epileptic should not the same be true for a psychopath with cerebral dysrhythmia? We help a person to correct or compensate for his inborn errors of metabolism; should this not also apply to someone who appears to have sexual urges too strong for him to control? The general rule in medicine is to help anybody with an organic complaint no matter how the condition arose, illnesses of enemy soldiers, accidents the result of mountaineering, excessive drinking, or promiscuity. These people are ill, with greater or lesser personality factors, but does social deviation without physical signs or symptoms come into the same category? The moment we enter the health or preventive field, we are faced with the problem of standards and values. If we keep to the curative field the onus is on the patient, he starts attending and ceases as he likes.

For myself, I can see no alternative between our accepting full responsibility for the individual and the gradual appearance of the social health expert who will be concerned with all the inter-personal and social aspects of a person and their family. The term "social deviant" is being used more and more. When Mack suggested Bristol as a good site for action research on Juvenile Delinquency, he, at the same time, queried whether there really was an entity Delinquency which could be dealt with as such. For him, Delinquency was an index of defective family relationships. In his monograph "Family and Community", he writes, "The mark of a well-established community is that there is comparatively little outright misbehaviour, what there is, is kept inside the group." He linked deprivation of loving care in early life with a crippled capacity to take part in social life and to make relationships with other persons in later life.

The term "social deviants" includes not only delinquents but other types of non-conformers, the problem families, and even some who "escape" into illness. At one end, they may have a very unusual physical constitution, at the other they may have been completely lacking any normal upbringing, illegitimate, passed from foster to foster home. As people they may seem extroverted, aggressive, acting out their hostilities, at the other end, indrawn, passive solitary individuals. Dr. John Spencer and the Social Project Team recommended that these individuals and families when identified should be grouped together and the community should then allocate special teams of social workers to help them. This is in sharp distinction to what others have advised, namely to scatter them so that they cannot influence each other adversely.

Some may consider that this problem is almost entirely due to the lowering of general moral standards or an unexpected consequence of the increasing benefits of the Welfare State. Others may feel that if people are controlled to the point where no crime exists, serious consequences would result for the whole of society. A few may see it as entirely the fault of psychiatry.

The problem of the "middle area" is also symbolised by the opening by the Prison Commissioners of the new prison in Buckinghamshire which is really an observation medical unit. Previously it was prison or Broadmoor—the latter under Health, the former under the Home Office. This is merely further evidence that the line between bad and mad can no longer be sharply drawn. The medical profession have long recognised partial responsibility, and recently the law has accepted the concept of diminished responsibility, but at present it is really only used where it is a case of a capital offence.

Returning to the children, the delinquents as a group are uneasy children, though by the time we see them their basic insecurity may be masked by an "I don't care" attitude. Often they seem superficially to have no sense of responsibility, to be in no way put out by what they have done. Our present British practice is to enclose each family in its own home, which puts a far greater responsibility on the parents. There is no-one else to whom the children can go when their parents, to them, seem lacking in understanding or insight; this does not arise when an extended family is housed together. Incidentally, many of the so-called attempted suicides are really people needing help and not able to find it. Two young teenagers whom I saw at the B.R.I. in the last fortnight, for taking an overdose, had very real problems and for over a year had been trying to get their parents at least to discuss their problems; finally in desperation they took an overdose. Ministers of religion are often not wanted where they are needed most, and the same is true of many teachers. It is only the doctor who regularly crosses the threshold and can be aware of what is going on. Of the sample of 25 cases, 18 of the children had had some professional contact with the family doctor, and I believe that some member of the average family sees the doctor every three months.

The alternative to our shouldering the load would seem to be the revival in modern garb of the parish visitor, a combination of health visitor, child care officer, school welfare officer, a community official whose special responsibility would be the "social deviants", with all the problems that this would create, including the return of the old body-mind division, something which we left behind when we stopped seeing "cases" and began dealing with individuals with problems.

What probably holds us back is the fact that medicine is based on the assumption that we have something—knowledge, skill—which the patient wants, and that he voluntarily comes to us. This has never really been the case so far as children are concerned, for they always have to convince their parents that there is something the matter before we see them. Child Guidance started with the school child, in part because of problems connected with learning in school, especially those due to low intelligence, and in part because the child was mixing with his peers, and signs of maladjustment, unnoticed or ignored by parents, were being seen by the school-teachers. Now we are specially concerned with another phase of development, an earlier one. Instead of intellectual backwardness we are dealing with social deviance. Incidentally, the medical profession is directly responsible, in a subtle way, for its increase. The handicapped child, the weakling, used to die but, with the drop in infant mortality, we have a corresponding increase in the number of substandard children whom any mother would find a handful because of the extra attention and emotional care that they need.

These children are not very difficult to spot and I would like to suggest that every child should be examined on his second birthday. A suitable scale would be that devised by Dr. Mary Sheridan and published as a Ministry of Health pamphlet where the child is assessed for:

(1) Posture and large movements.

(2) Vision and fine movements.

(3) Hearing and speech.

(4) Social behaviour and play.

This scale gives norms for each three months of development.

If the child is more than six months retarded in any one of these divisions, then I would further suggest that the home be rated for its ability to meet that particular child's needs. If there seems a high degree of probability that those needs, whether physical or emotional, will not be met, it would then be for the community, guided by the family doctor, to decide what special steps should be taken. By such an examination, not only would physical handicaps be spotted earlier and appropriate remedial measures taken, but the chances of social deviance would be lessened and juvenile delinquency, being one of the commoner outcomes, could perhaps be averted.

